# Low-cost synthesis of peptide libraries and their use for binding studies via temperature-related intensity change

**DOI:** 10.1016/j.xpro.2021.100605

**Published:** 2021-06-15

**Authors:** Clemens Schulte, Vladimir Khayenko, Amit Jean Gupta, Hans Michael Maric

**Affiliations:** 1 Rudolf Virchow Center, Center for Integrative and Translational Bioimaging, University of Wuerzburg, Josef-Schneider-Str. 2, 97080 Wuerzburg, Germany; 2 Biocenter, Department of Biotechnology and Biophysics, University of Wuerzburg, 97074 Wuerzburg, Germany; 3 Nanotemper Technologies GmbH, Flößergasse 4, 81369 Munich, Germany

**Keywords:** Biophysics, Biotechnology and bioengineering, High Throughput Screening, Molecular/Chemical Probes, Protein Biochemistry

## Abstract

Protein-peptide interactions are involved in many fundamental cellular functions and constitute promising drug targets. Here, we provide a detailed protocol for the cost-effective preparation of a cellulose-based solid support for synthesis of nanoscale to micromolar-scale peptide libraries. Their subsequent use for high-throughput protein interaction screening as well as affinity determination in solution provides binding data for thousands of unique peptides with a turnover of 1 to 2 weeks, thereby facilitating *in vitro* assessment and development of high-affinity binders.

For complete details on the use and execution of this protocol, please refer to Schulte et al., (2020)

## Highlights

• Facile and cost-effective preparation of a cellulose-based solid support
• Automated peptide synthesis on cellulose disks after laser perforation
• Seamless transition from peptide synthesis to the binding assay
• Quasi-label-free protein affinity determination in solution

## Graphical abstract

**Figure:**
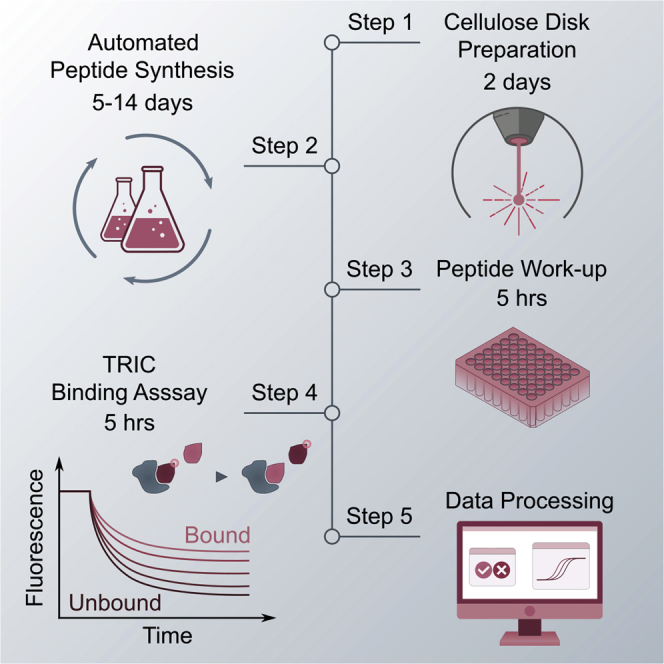

## Before you begin

Here, we provide a cost-effective approach for high-throughput studies of protein-peptide interactions, specifically with temperature-related intensity change (TRIC) measurements using the Dianthus NT.23 system (Nanotemper Technologies GmbH). Using state of the art peptide chemistry and the currently available materials, we also provide an updated version of the popular cellulose-based SC^2^ (Dikmans et al., 2006, McBride et al., 2016) and SPOT (Frank, 1992, Mao et al., 2021) approaches. The protocol is tailored for the automated and parallel peptide synthesis with the MultiPep 2 (CEM GmbH) or similar liquid handling systems and scaled towards the need of the high-throughput affinity determination in solution. Laser cutting to minimize hands-on time and cost of solid support preparation and quality control using mass spectrometry are highly recommended but optional. The protocol combines synthesis and evaluation, maintaining the cost-effectiveness of the widely available cellulose-based peptide synthesis approaches and their seamless transition into in-solution TRIC measurements.

### Preparation of functionalized cellulose disks

**Timing: 1 day**

Functionalized cellulose disks are commercially available. However, if there is access to a laser cutting device (key resources table), we recommend in-house preparation. The functionalized cellulose will serve as a solid support for the automated peptide synthesis and is a cost-efficient alternative to commercially available solutions, having the additional advantage that the degree of functionalization may be fine-tuned by varying the reaction time and composition of the functionalization mix.

1. Cut the Whatman paper sheets into pieces of appropriate size (15 × 10 cm) using a paper trimmer.
2. Dry the paper under vacuum in a desiccator for at least 8 h.
  a. To simplify handling, wrap the sheets into an envelope made of Kimtech wipes.
3. Prepare functionalization mix (sufficient for 6 sheets).
  a. 3.7 g Fmoc-β-Ala-OH, 2.3 mL N,N'-Diisopropylcarbodiimide (DIC), 0.96 mL 1-Methylimidazole (NMI).
  b. Add the Fmoc-βAla-OH to 50 mL Dimethylformamide (DMF) and fully dissolve, then add DIC/NMI and fill to 60 mL with DMF. End concentrations: Fmoc-βAla-OH 0.2 M (1 eq), DIC 0.24 M (1.2 eq), 0.2M NMI (1 eq).

***Note:*** Reducing the degree of Fmoc-β-Ala-OH functionalization by employing a functionalization mix with equal amounts of Fmoc-β-Ala-OH and Boc-Gly-OH can largely improve synthesis yields for long (20-30AA) or aggregation-prone sequences (Problem 1).

4. In a fume hood, Incubate the sheets for 3 h individually in 10 mL of the functionalization mix, using a flat plastic container (Raaco, part no.: 136129).
  a. Place the plastic containers on an orbital shaker rotating at ∼60 rpm
  b. After 1.5 h, flip the sheets using a metal tweezer.
5. Discard the functionalization mix and wash the sheets with 3 × 10 mL DMF for 1 min each, followed by 3 × 10 mL EtOH for 1 min each.

***Note:*** When discarding the DMF or EtOH, hold the paper sheet in place with a metal tweezer.

6. Let the EtOH evaporate for ∼15 min in a fume hood.
7. Finish drying in a desiccator for at least 8 h.

### Membrane laser cutting

**Timing: 3 h**

In this step, the functionalized cellulose is cut using a laser to fit into 384-format trays (Methods video S1). The here presented settings were optimized for a Sabko SH-G460 80-Watt CO_2_ laser.

8. Settings
  a. 100 mm/s cutting speed, low speed reduces mechanical stress.
  b. Power setting >4 W to ensure stable operation.
  c. Zinc selenide focusing lens with 2” focal length.
  d. Total cutting time: 200 s for 600 pcs.
9. Final product properties.
  a. Cut width: 0.1–0.2 mm.
  b. Disk diameter: 3.6 mm.

***Note:*** The settings for cutting should be chosen so that the cellulose sheets are perforated in a way that allows for convenient transfer of the resulting disks to the 384-well plates. Cutting the disks out completely is possible but they would need to be transferred using a tweezer, thereby significantly increasing hands-on time (Methods video S1).

Methods video S1. Laser-based perforation of functionalized cellulose solid-support Laser strength is adjusted so that the paper sheet is perforated but individual disks are not fully removed to create paper-disk templates that enable convenient handling and transfer to 384-format trays. Related to (step 8).

### Validating Fmoc-β-Ala-OH loading

**Timing: 1 h**

Measuring the Fmoc-β-Ala-OH loading is critical to determine how much peptide can be expected to be synthesized on each disk. This will ultimately determine the number of disks required to synthesize a certain amount of peptide and also influence the efficiency of peptide elongation. Here, the concentration of the Fmoc protection group is measured after deprotection of a single disk to determine the loading.

10. From each batch of functionalized cellulose membrane (six sheets), at least six disks (a triplicate from two sheets) should be used for determination of Fmoc-β-Ala-OH loading.
11. Transfer each disk to an individual reaction tube.
12. Add 1 mL of 20% (v/v) piperidine (pip) in DMF per tube.

***Note:*** Prepare an excess of this solution since the same solution will be used for calibrating the spectrophotometer for the measurement and cleaning the cuvette.

13. Perform Fmoc deprotection in a thermoshaker for 20 min at 500 rpm and ambient conditions.
14. Dilute 500 μL of each deprotection solution from each tube with 500 μL of 20% pip in DMF in a new reaction tube.

***Note:*** If the Fmoc concentration of the solution after deprotection is higher or lower than your spectrophotometer allows for, account for this by adjusting the dilution factor.

15. Calibrate the absorbance of the spectrophotometer at 290 nm using 20% pip in DMF.

***Note:*** clean your cuvette with the same 20% pip in DMF solution prior to the calibration to avoid contamination.

16. Measure the absorbance three times for each solution and consider the average value for determination of the Fmoc-β-Ala-OH loading.
  a. Use equation (1) to determine the Fmoc concentration and thus the degree of Fmoc-β-Ala-OH loading in nmol.

(Equation 1) $$Loading=\left(\frac{\bar{A}}{6.1206}\cdot DF\right)\cdot PipVol\cdot 10^{6}$$

With

$\bar{A}$: average absorbance at 290 nm

DF: dilution factor (2 in this case)

PipVol: Volume of pip solution in mL (1 in this case)

***Note:*** The here described functionalization protocol should result in an Fmoc-βAla loading of ∼100 nmol/disk.

**CRITICAL:** Due to a high inter-batch variability of up to 50%, it is necessary to determine Fmoc-β-Ala-OH loading for each batch of functionalized cellulose prepared. In our experience, an intra-batch variability of 20% is acceptable.

### Peptide library design

Critical aspects that must be considered when designing the peptide library are:

17. Amount of each peptide required to characterize the interaction.
  a. This depends on the expected K_D_ value of the protein-peptide interaction. For a binary screening/mapping, the amount should be selected so that the peptide concentration in the final assay buffer volume (see “peptide work-up”), exceeds the expected K_D_ value by a factor of 100.
  b. The amount of each peptide may be adjusted by the number of disks that the respective peptide is synthesized on. In our experience, protein-peptide interactions with an affinity in the low μM range can be studied with peptides synthesized on two disks with an average Fmoc-β-Ala-OH loading of 130 nmol. In our case, the peptides were solubilized in 50 μL of assay buffer to reach an end concentration of 500–700 μM.
18. Quality Control.
  a. To assess the peptide purity after synthesis, extra peptides should be accounted for when designing the library. We recommend adding 6 peptides on each 384 plate for quality control purposes. In this exemplary case, internal duplicates of 3 random peptides from the library are included in each 384 plate to account for possible variations in the synthesis.
19. Layout of the peptides in 384-format.
  a. Since the synthesis is performed in 384-well format and the work-up takes place in 96-well format, the initial peptide layout should be designed in a way that allows for convenient transfer of the disks from one format to the other. We recommend choosing the layout so that the same peptide sequence in synthesized in adjacent wells.
20. Include an additional linker between the cellulose solid support and a moiety that allows for cleavage of the peptides under acidic conditions (Figure 1).
  a. We recommend introducing an additional βAla linker (“B”) before synthesizing the peptides of interest.
  b. To facilitate cleavage of the peptides after the synthesis using standard trifluoracetic acid (TFA) cleavage conditions (see Peptide Work-up), a rink-amide linker (“1”) is introduced before the peptide chain is synthesized. The protocol may be adapted to include any linker for standard Fmoc solid-phase peptide synthesis (SPPS) that allows for peptide cleavage under similar conditions.

**Figure 1:**
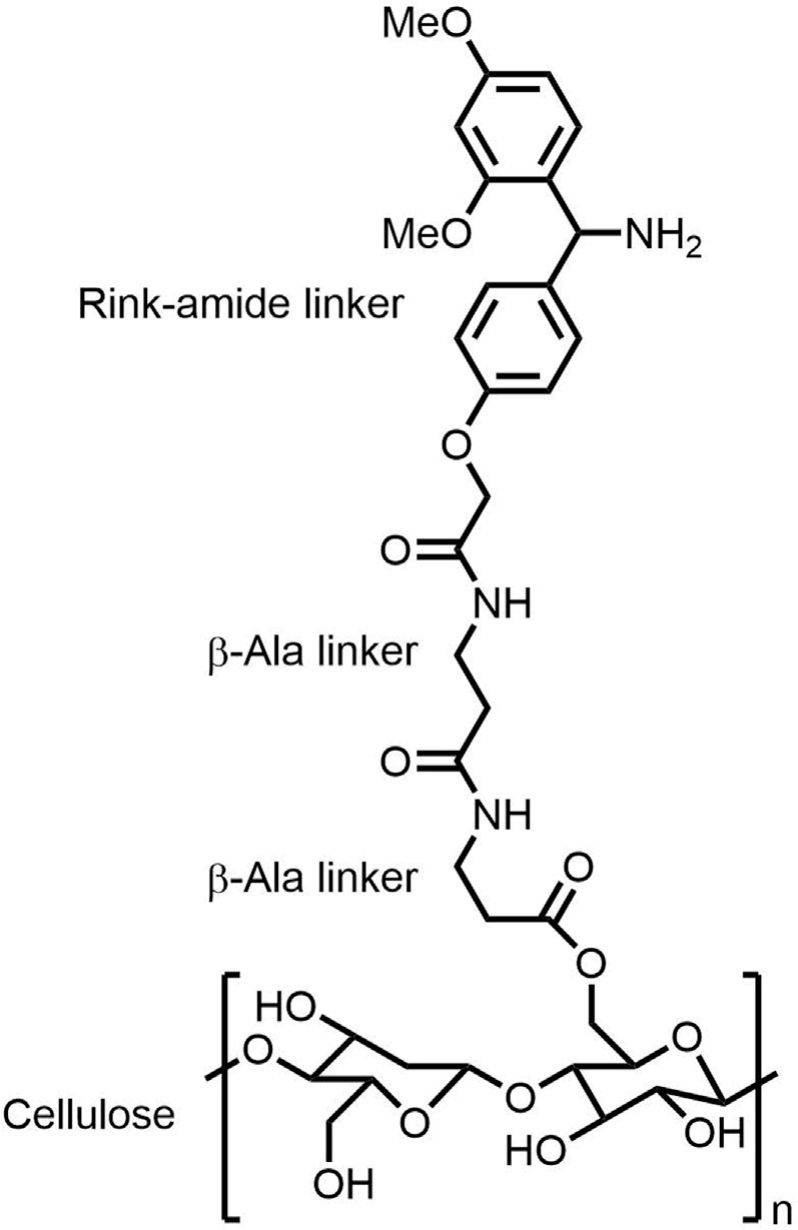
Schematic representation of the modified paper support The cellulose support is functionalized with two β-Ala linkers and a Rink-amide linker for peptide cleavage.
21. Peptide length.
  a. Ideally, all peptides synthesized should contain the same number of amino acids (aas) due to the lack of a universal purification step. Affinity data obtained with peptides of largely differing length can be expected to be biased by variations in amounts and purities. In this line, we recommend an upper limit of 30 aas for affinity determination, since a commonly observed coupling efficiency of 93%–98% corresponds to a peptide purity of 11%–55% (Schulte et al., 2020, Moreno-Yruela et al., 2020, Sereikaite et al., 2019) . Generally, representative quality controls should always be included in any peptide library to assess the peptide purity (see Quality Control by LC-MS).

### Preparation of aa derivates and reagents

**Timing: 2 h**

The here presented protocol encompasses standard SPPS using DIC and Oxyma for coupling, which exhibit higher stability over time than other coupling reagents such as COMU (El-Faham and Albericio, 2010) and are easier to handle than HOBT (Subirós-Funosas et al., 2009). Due to inadequate half-life for automated synthesis, we recommend to employ acetic anhydride for capping without an additional, sterically hindered base (Pon et al., 1999) but longer incubation steps and repetition.

22. Prepare stock solutions of the aa building blocks at a concentration of 0.5 M in DMF.

***Note:*** Certain aas, especially Fmoc-Pro-OH and Fmoc-Phe-OH, might need extra agitation to fully dissolve. We recommend dissolving these derivates on an orbital shaker for 1 h at ambient conditions.

23. Prepare solutions of DIC and OxymaPure at a concentration of 1 M in DMF and acetic anhydride (Ac_2_O) at 4% (v/v) in DMF

***Note:*** Since these solutions need to be exchanged every 48–72 h, we recommend preparing 15 mL of DIC and Oxyma stock solutions and 30 mL of Ac_2_O stock solution each time in a 50 mL falcon tube.

24. Prepare 180 mL of a deprotection solution containing 20% (v/v) pip in DMF
  a. Commonly, more than 180 mL (the max volume of reagent containers in a MultiPep 2 synthesizer) are needed for one synthesis. Therefore, it is advised to pay attention to the amount of deprotection solution left in the container during synthesis and refill it accordingly.

***Note:*** We recommend preparing the aa stock solutions in advance. The reagents however should be prepared on the same day as the synthesis is started.

**Pause point:** Stock solutions of aa derivates in DMF can be stored at −80°C for several months.

### Setting up the MultiPep 2 synthesizer

**Timing: 2 h**

These steps are necessary to be carried out before each synthesis.

**Figure 2:**
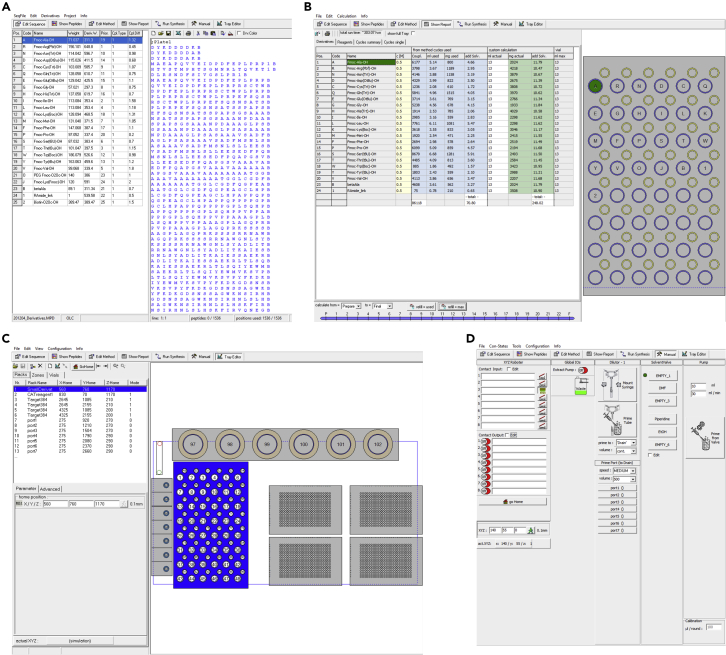
Overview of the MultiPep 2 – CelluSpots software Shown are screenshots from the MultiPep 2 – CelluSpots program. (A) Derivatives (left) and Sequences (right) in the MultiPep 2 - CelluSpots program. Parameters such as a one letter abbreviation and the respective molecular weight can be specified for each aa derivative used. On the right, the peptide sequence table is entered. (B) Exemplary report sheet in the MultiPep 2 – Celluspots program. The required volume (left) and the position in the derivatives rack (right) are given for each aa derivative. (C) Tray settings in the MultiPep 2 – CelluSpots program. X, Y and Z position value settings for each position (left) and a schematic model of the MultiPep 2 robot for maneuvering of the needle (right). (D) Manual settings of the MultiPep 2 – CelluSpots program. Here, the needle that is used for liquid handling can be primed.

***Note:*** The Multipep 2–CelluSpots software contains the parameters of certain aa derivatives by default, such as the 20 proteogenic aas. Mass and names of additional derivatives such as non-proteinogenic aas or the shown Biotin-O2O-OH building block (Figure 2A) need to be entered manually. The upper limit of building blocks is limited by the rack for 10 mL tubes (our setup harbours 48 slots).

***Note:*** after entering the peptide sequences, we recommend making use of the “cleanup sequences” and “check sequences” feature.

***Note:*** Make sure to save and archive the sequence tables in the proprietary .SEQ format.

25. Enter the peptide sequences of interest the MultiPep 2 software (Figure 2A).

26. Create a report sheet to determine the required volume of each building block (Figure 2B).
  a. Switch to “Show Report”.
  b. Click “Refill=Used” to calculate the required volumes.

***Note:*** Always add the volume stated under “mL actual” to the 10 mL reaction tubes. In this column, the dead volume is considered.

***Note:*** The report sheet can be printed to .pdf format under “File”–“Print Report Sheet”.

27. Add the amounts of aa building blocks that are indicated in the report sheet to proprietary 10 mL reaction tubes.
28. Place the 10 mL reaction tubes and 2 mL mixer tubes to the indicated positions in the reagent rack.

***Note:*** 10 mL reaction tubes can be cleaned and re-used after the synthesis. We recommend discarding the 2 mL mixer tubes after the synthesis.

29. Empty the waste container that is connected to the vacuum pump if necessary.
  a. Ensure an airtight connection of the waste container to the vacuum pump. This can be tested by running the vacuum pump after closing the connection.
30. Connect the 384-format rack to the vacuum pump (Figure 3).

**Figure 3:**
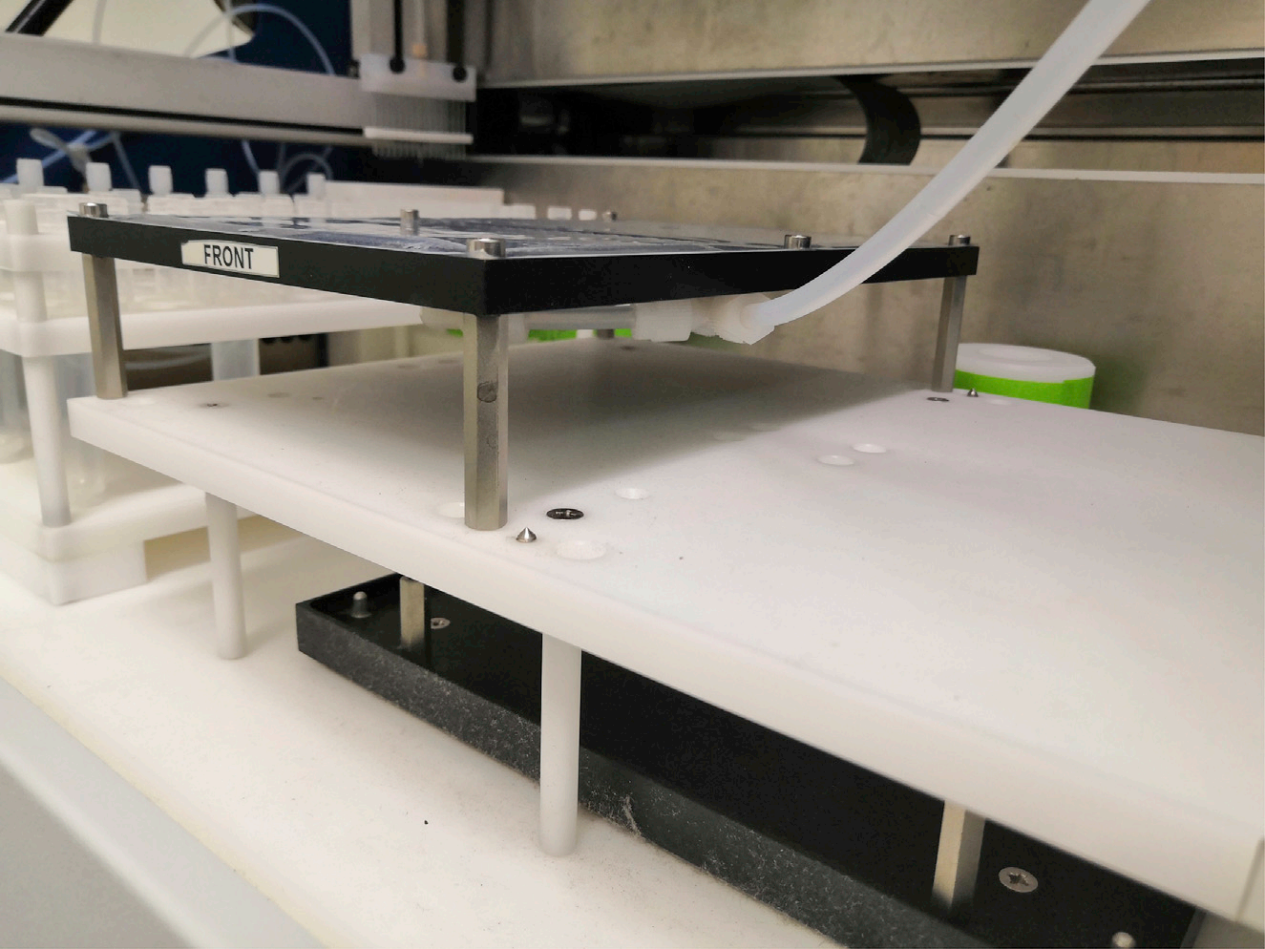
Proper tray holder connection Ensure a tight connection between the vacuum pump tubing and the tray holding the 384-well plates.
31. Transfer functionalized cellulose disks to 384-format trays (Figure 4) and place the trays in the rack.
  a. Ensure proper positioning of the cellulose disks in the 384-well plate. Loose disks could be removed from their wells by the robot during synthesis.
  b. Make sure to properly close the close the rack to ensure vacuum suction during the synthesis.
  c. If only one 384-well plate is being used for synthesis, load another, empty plate into the second position on the rack and close it with a 384-well plate silicon sealing mat (CEM) to avoid loss of vacuum.

**Figure 4:**
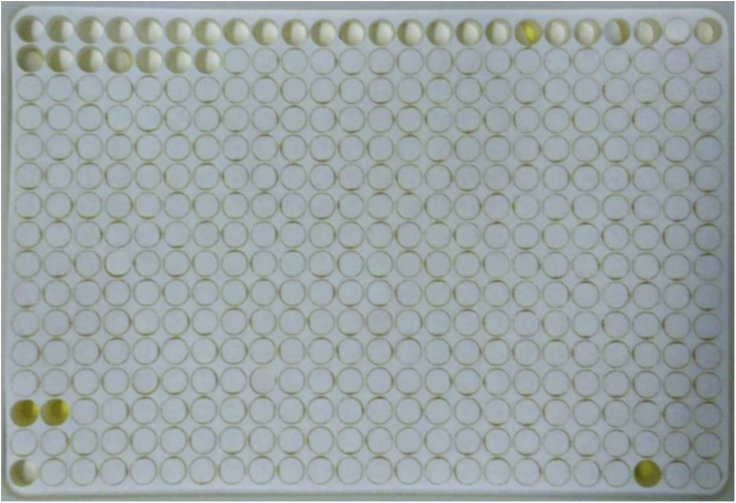
Perforated disk sheet Representative example of a functionalized and perforated cellulose sheet placed onto a 384-well tray for facilitated transfer of individual disks.
32. Fill the reservoirs for DMF and EtOH that are used for washing.
33. Place the reservoirs for Pip, DIC, Oxyma and Ac_2_O in the respective positions indicated in the Method section of the MultiPep 2 – CelluSpots program (see report sheet).
34. Control the x/y/z-positioning of the robot arm by manually driving it to the corner positions of each 384-well plate (Figure 2C) (Problem 2).
  a. Manually select a corner well of a 384-well plate.
  b. Maneuver the needle to the corresponding position using the “go to” command.

***Note:*** If the needle is not properly positioned in the well, adjust the X, Y, or Z values. Note that increasing the X-values will move the robot more arm more to the right, increasing the Y-values will more it more to the front of the robot and increasing the Z-value will move it more downwards, *vice versa*.

35. Prime the syringe with DMF and EtOH in the “Manual” section of the MultiPep 2 – CelluSpots program (Figure 2D).

**CRITICAL:** All air bubbles need to be removed from the tubing connecting the needle to the DMF and EtOH reservoir during priming.

## Key resources table

**Table:** 

REAGENT or RESOURCE	SOURCE	IDENTIFIER
**Chemicals, peptides, and recombinant proteins**		

DMF (for peptide synthesis)	Iris Biotech	SOL-004
OxymaPure (Novabiochem)	Iris Biotech	RL-1180
DIC	Iris Biotech	RL-1015
Piperidine PEPTIPURE®	Carl Roth	A.122
Ac_2_O ROTIPURAN®	Carl Roth	CP28
Fmoc-β-Ala	Iris Biotech	FAA1300
Fmoc-Rink Amide-Linker	Iris Biotech	RL-1027
Fmoc-L-Ala-OH·H2O	Iris Biotech	FAA1005
Fmoc-L-Cys(Trt)-OH	Iris Biotech	FAA1040
Fmoc-L-Asp(tBu)-OH	Iris Biotech	FSC1020
Fmoc-L-Glu(tBu)-OH·H2O	Iris Biotech	FSC1045
Fmoc-L-Phe-OH	Iris Biotech	FAA1175
Fmoc-Gly-OH	Iris Biotech	FAA1050
Fmoc-L-His(Trt)-OH	Iris Biotech	FAA1090
Fmoc-L-Ile-OH	Iris Biotech	FAA1110
Fmoc-L-Lys(Boc)-OH	Iris Biotech	FAA1125
Fmoc-L-Leu-OH	Iris Biotech	FAA1120
Fmoc-L-Met-OH	Iris Biotech	FAA1150
Fmoc-L-Asn(Trt)-OH	Iris Biotech	FAA1015
Fmoc-L-Pro-OH·H2O	Iris Biotech	FAA1185
Fmoc-L-Gln(Trt)-OH	Iris Biotech	FAA1043
Fmoc-L-Arg(Pbf)-OH	Iris Biotech	FAA1010
Fmoc-L-Ser(tBu)-OH	Iris Biotech	FAA1190
Fmoc-L-Thr(tBu)-OH	Iris Biotech	FSC1210
Fmoc-L-Val-OH	Iris Biotech	FAA1245
Fmoc-L-Trp-(Boc)-OH	Iris Biotech	FAA1225
Fmoc-L-Tyr(tBu)-OH	Iris Biotech	FAA1230
NMI	Sigma-Aldrich	M50834
TFA (≥99.9%, for peptide synthesis)	VWR	84578.290
DCM (≥99.8%)	Sigma-Aldrich	270997
TIPS (98%)	Sigma-Aldrich	233781
Diethyl ether (anhydrous, ≥99.7%)	Sigma-Aldrich	296082
Pluronic F-127 (BioReagent)	Sigma-Aldrich	P2443
NaCl (p.a., ACS, ISO)	Carl Roth	231-598-3
KCl (p.a., ACS, ISO)	Carl Roth	231-211-8
Na_2_HPO_4_ (≥99%, p.a., ACS)	Merck	1.065.86.0500
KH_2_PO_4_	VWR	26936.260
L-Glutathione reduced (≥98.0%)	Sigma-Aldrich	G4251

**Software and algorithms**		

DI.Screening Analysis	NanoTemper Technologies GmbH	DI-S001S
DI.Control	NanoTemper Technologies GmbH	N/A
MultiPep 2 - CelluSpots	CEM GmbH	N/A

**Other**		

MultiPep 2 Synthesizer	CEM GmbH	N/A
Dianthus NT.23PicoDuo	NanoTemper Technologies GmbH	N/A
NanoDrop^TM^ spectrophotometer	Thermo Fisher Scientific	ND2000CLAPTOP
Thermoshaker with cooling	Thermo Fisher Scientific	12357627
Whatmann filter paper 50 (hardened, low ash)	Sigma-Aldrich	WHA1450916
Sabko SH-G460 80-Watt CO2 laser	Sabko GmbH	N/A
Precision XS liquid handling robot	BioTek	N/A
STARlet liquid handling robot	Hamilton	N/A
96-Deepwell plates	VWR	737-2510
384-Well Dianthus plate	NanoTemper Technologies GmbH	DI-P001A
Assorter polypropylene boxes	Raaco	136129
Kimtech Science Precision Wipes	Kimtech Science	7552
13 mL Tubes	Intavis Bioanalytical Instruments AG	32.035
2 mL Screw car mixing vials	Intavis Bioanalytical Instruments AG	35.202

## Materials and equipment

**Table:** PBS

Reagent	Final concentration [mM]	Amount
NaCl	137	8 g
KCl	2.7	0.2 g
Na_2_HPO_4_	10	1.78 g
KH_2_PO_4_	1.8	0.24 g
H_2_O	N/A	1 L
Total	N/A	1 L

Store at 4°C for up to 2 months

**Table:** TRIC assay buffer

Reagent	Final concentration	Amount
PBS	N/A	99.9 mL
Pluronic F127	0.1%	100 μL
L-Glutathione (reduced)	2 mM	61.46 mg
Total	N/A	100 mL

Store at 4°C for up to 2 weeks

***Alternatives:*** This protocol describes the in-house preparation of functionalized cellulose solid support for solid phase peptide synthesis including the perforation of the paper using a laser cutting device., The paper disks can be bought from commercial sources (Intavis Peptide Services GmbH & Co. KG). Instead of a laser cutter, disks with a diameter of 3.6 mm can be mechanically punched out.

***Alternatives:*** N-Methyl Pyrrolidone (NMP) can be used as a solvent for aa derivates that exhibit insufficient solubility in DMF.

***Alternatives:*** Here, we describe automated peptide synthesis in a MultiPep 2 robot (CEM GmbH). In principle, the synthesis may be carried out in any liquid handling robot that allows for SPPS using low μL volumes.

## Step-by-step method details

### Peptide synthesis

**Timing: 5–14 days**

The automated peptide synthesis in the MultiPep 2 robot follows a standard Fmoc chemistry procedure. Timing may vary strongly based on the length of the desired peptides.

**Figure 5:**
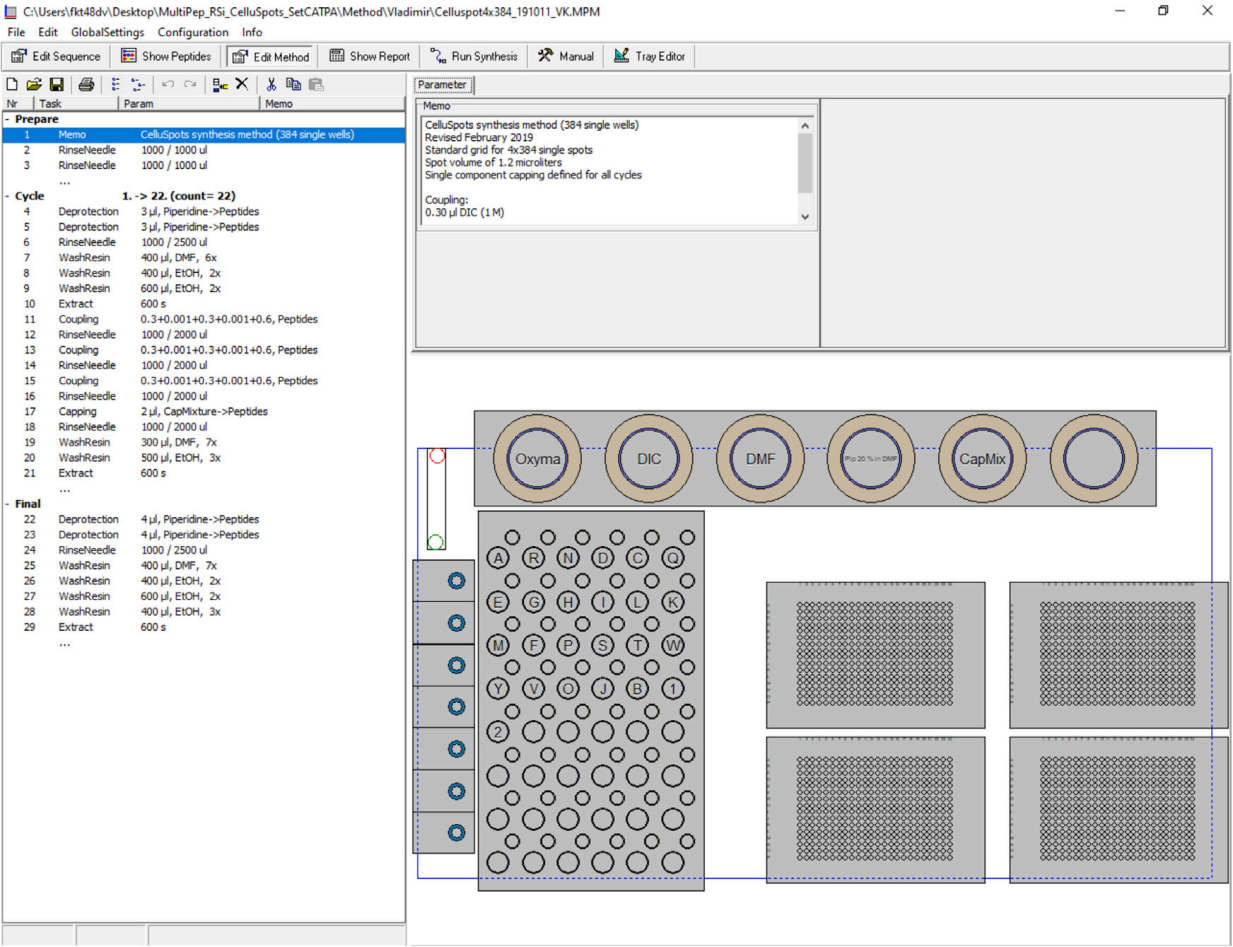
Methods settings in the MultiPep 2 – CelluSpots software Preparation, Cycle and Finalization steps (left) and schematic depiction of the robot tray (right).

1. Set up the MultiPep 2 synthesizer to carry out the steps described in Table 1 until the desired peptides are generated.
  a. The synthesis method can be adjusted in the “Methods” section of the program (Figure 5).

**Table 1:** Automated SPPS coupling cycle

No.	Action	Reagent	Solvent	Vol. [μL]	Time [min]	Repeats
1	Deprotection	20% (v/v) Pip	DMF	3	10	2×
2	Wash	-	DMF	400	-	6×
3	Wash	-	EtOH	400	-	2×
4	Wash	-	EtOH	600	-	2×
5	Extract	-	-	-	10	-
6	Coupling	1 M DIC 1 M Oxyma 0.5 M aa derivative	DMF	1.2	30	3×
7	Capping	4% (v/v) Ac_2_O	DMF	2	5	-
8	Wash		DMF	300	-	7×
9	Wash		EtOH	500	-	3×
10	Extract		-	-	10	-

All concentrations are given as final.

***Note:*** We generally recommend performing three coupling steps for each aa and two Fmoc-deprotection steps, independently of parameters such as peptide sequence or length. The upper limit of the coupling time is determined by the evaporation of the reaction mix. Here, we use 1.2 μL, which allows for coupling times of up to 30 min.

**CRITICAL:** Change the DIC, Oxyma Pure and Ac_2_O solutions every 48–72 h. This also goes for aliquots of Fmoc-Phe, Fmoc-Pro and Fmoc-His, which tend to form percipitates upon evaporation of the solvents.

2. Finalize the synthesis with the steps described in Table 2.

**Table 2:** Completion of automated SPPS

No.	Action	Reagent	Solvent	Vol. [μL]	Time [min]	Repeats
1	Deprotection	20% (v/v) Pip	DMF	4	10	2×
2	Wash		DMF	400	-	7×
3	Wash		EtOH	400	-	2×
4	Wash		EtOH	600	-	2×
5	Wash		EtOH	400	-	3×
6	Extract		-	-	10	-

### Peptide work-up

**Timing: 6 h**

Side chain deprotection and concomitant cleavage are performed with TFA and scavengers. The disks must be fully dried before deprotection is started.

3. Prepare cleavage and sidechain-deprotection mix.
  a. 90% TFA, 2% dichloromethane (DCM), 5% H_2_O, 3% triisopropylsilane (TIPS).
  b. Prepare a 10% excess to account for evaporation.
  c. Mix well and gradually release the pressure from the bottle.
4. Add 150 μL cleavage and sidechain-deprotection mix to each well with a stepper-pipette.
  a. Make sure to properly cover the disks as they may adhere to the side of the deepwells due to electrostatic attraction.
5. Incubate the disks on an orbital shaker at 1,000 rpm for 3 h.
6. Transfer the solution to new 96-deepwell plates.
  a. When multichannel pipettes are used, we recommend the use of filtered tips to avoid internal damage.
7. Add 700 μL ice cold ether per well to precipitate the peptides.
8. Incubate the deepwell plates at −20°C for 8 h.
9. Centrifuge the plates at 2,000 × *g* for 30 min and 4°C.
  **CRITICAL:** Check if a pellet is visible in the deepwells. Depending on the amount of peptide prepared, an additional centrifugation at 2,000 × *g* for 30 min might be necessary. Handle gently to avoid discarding the peptide pellets along with the supernatant.
10. Gently discard the supernatant and again, add 700 μL ice cold ether to each well.
11. Agitate the plates on a vortex mixer to fully dispense the pellet.
12. Centrifuge the plates at 2,000 × *g* for 30 min and 4°C.
13. Gently discard the supernatant and let the ether evaporate in a fume hood for 15 min or until the pellet is visibly dry (Problem 3).

**Pause point:** Cleaved peptides may be stored at −20°C for at least one month. For storage, close the 96-deepwell plates with silicone sealing mats.

### Quality control by LC-MS

**Timing: 8 h**

To assess the peptide purity after synthesis, which may vary depending on peptide length and sequence, we recommend conducting liquid chromatography coupled to mass spectrometry (LC-MS) measurements of control peptides (Schulte et al., 2020, Moreno-Yruela et al., 2020, Sereikaite et al., 2019) (see 18). Alternatively, matrix-assisted laser desorption-ionization-time of flight (MALDI-TOF) mass spectrometry can be performed to determine peptide purity.

14. Transfer the disks with the quality control peptides to an individual reaction tube.
15. Add 150 μL of cleavage and sidechain-deprotection mix to each reaction tube and incubate in a thermoshaker at 1000 rpm for 2 h at ambient conditions.
16. Transfer the cleavage and sidechain-deprotection mix to new reaction tubes.
17. Add 1 mL of ice-cold ether to each reaction tube and incubate for 2 h at −20°C
18. Centrifuge the reaction tubes at 15,000 × *g* and 4°C for 15 min.

**CRITICAL:** Check if a pellet is visible (Problem 3) in the reaction tubes and handle the tubes carefully to not disturb the pellet.

19. Discard the supernatant and add 1 mL of ice cold ether
20. Vortex the reaction tubes thoroughly and centrifuge again at 15,000 × *g* and 4°C for 15 min
21. Discard the supernatant and repeat the washing step as before.
22. Let the ether evaporate in a fume hood for 15 min or until the pellet is visibly dry.
23. Dissolve the peptide by adding 100 μL of a buffer consisting of 49.95% H_2_O, 49.95% acetonitrile and 0.1% formic acid and incubating in a thermoshaker at 1000 rpm for 1 h at ambient conditions.
24. Filter the peptide solution through a 0.45 μM pore-size PVDF filter and use for LC-MS purity assessment.

***Note:*** Depending on the LC-MS or MALDI-TOF system available, sample preparation may need to be varied.

### TRIC measurements

**Timing: 1 day**

While in most cases, TRIC measurements are conducted using fluorescently labeled proteins (Asmari et al., 2018), we present a protocol for quasi-label free measurements using a fluorescent tracer, which prevents the need to fluorescently label the protein of interest.

When employing a fluorescently labeled target, it is critical to determine the stability of the labeled protein before performing the TRIC assay and to potentially adjust the parameters such as the labeling strategy or the assay buffer (Toseland, 2013).

25. Add 50 μL/well TRIC assay buffer to the 96-well plates containing the dried peptides.
26. Shake the 96-well plates for 1 h at ambient conditions to solubilize the peptides.

***Note:*** Solubility can differ substantially between different peptides in your library, so the time for solubilization might need to be adjusted.

27. Centrifuge the 96-well plate at 1,000 × *g* for 15 min to remove precipitate.
28. Determine the concentration of each peptide using a NanoDrop (Thermo Fisher Scientific).
  a. Peptide concentrations can be easily quantified using the Scopes method (Scopes, 1974). We furthermore recommend to account for peptide sequence-specific differences in absorbance (https://spin.niddk.nih.gov/clore/) (Anthis and Clore, 2013). Peptide concentrations may further be determined by high performance liquid chromatography (HPLC).
29. Using a liquid handling robot, transfer the peptide solutions with known concentration into a conventional 384-well plate. In this case we used a PrecisionXS liquid handling system (Biotek).
  a. At this point, a dilution step can be implemented to adjust the concentration of your peptides in accordance to the expected K_D_ value of the interaction between the peptides and the target protein. We diluted our library of unlabeled peptides 2:1 in TRIC assay buffer.

**CRITICAL:** Depending on the type of assay to be conducted, transfer the peptides either along a 12-point serial dilution to perform a dose response (Dose response affinity determination) or at equal concentrations for single-dose measurements (Single-dose screening).

***Note:*** TRIC can resolve affinities ranging from low picomolar to millimolar dissociation constants. The resolvability of weak affinities is only limited by the solubility of the titrated molecule at high concentrations. Resolving strong affinities in the picomolar range is limited by the affinity of the fluorescently labelled tracer in displacement assays.

**CRITICAL:** The affinity between tracer and target must first be determined in a 1:1 binding experiment in order to derive K_**i**_ values from dose response experiments and furthermore to determine the optimal concentrations of tracer and target. In our case, a K_**D**_ value of 1.54 nM was determined, based on which we employed the target protein and fluorescent tracer at a concentration of 20 nM and 10 nM, we generally recommend using a low nanomolar concentration of tracer and a concentration of target protein of 1–2**×K**_**D**_ (of the tracer-protein complex). The lower end of the affinity range that can be resolved will approximately correspond to the K_**D**_ of the tracer-protein complex, the higher end will be limited by the concentration of the protein target. Interestingly, opposite to common assumption, high-affinity tracer molecules do work well for competition assays **(****Huang, 2003****)**. The higher the affinity of the tracer, the broader the range of affinities that can be resolved in competition assays. However, high affinities are usually associated with slow binding kinetics. Hence, long incubation times are recommended for tracer molecules with low nanomolar to picomolar affinities to the protein target. A simple control experiment, where the labelled tracer is displaced by the same unlabeled ligand molecule, can be used to test if a system reaches equilibrium after selected timepoints.

30. Using a liquid handling robot, mix the unlabeled peptide ligands with the pre-incubated tracer-target complex solution in a 384-well Dianthus plate and incubate at 4°C.
  a. Adjust the incubation time of the tracer with the unlabeled ligand, depending on the expected kinetics of the studied protein-peptide interaction. In our case, incubation was performed for 16 h to ensure the system had sufficient time to reach kinetic equilibrium.
31. Immediately before measuring, centrifuge the 384-well Dianthus plate at 1,000 × *g* for 30 sec. (Problem 4)
32. Set the set temperature of the Dianthus NT.23 PicoDuo system to 25°C and ensure that the prepared plate has sufficient equilibration time to also reach 25°C. This step ensures temperature homogeneity of all wells during the 30 min read time required to measure one full plate.

***Note:*** Assay temperature should be adjusted according to the protein-peptide interaction of interest, specifically the proteins thermal stability.

33. Perform the measurement with optimal parameters. Parameters should be selected depending on characteristics of the fluorophore and interaction type.
  a. Depending on the concentration of fluorescent tracer molecule, the LED power of the instrument should be set to reach between 10,000 and 15,000 counts for the reference sample.
    ***Note:*** While performing a screen, the setting should not be set to auto but given a fixed value that is consistent for all plates measured in one screen.
  b. The IR-LASER on-time should be selected based on the assay development measurements. For most assays, an on-time of 5 sec is recommended. Only for large assays with many ligands, an on-time of 3 sec is recommended to reduce measurement time and increase throughput.

### Data analysis (single-dose screening)

**Timing: 30 min**

34. In DI.Control, a reference ID is assigned to each well of the measured assay.

***Note:*** References (target-only) controls are needed to compare the signal obtained in presence of various ligands. We generally recommend placing references according to the liquid handling system used. In the present case, a 4-channel STARlet system (Hamilton) was used. Hence, references were placed in each pipetted row in duplicate and ligand wells were always grouped with references that were prepared with the same pipettor.

35. All relevant files are loaded into DI.ScreeningAnalysis and the relevant experiments are selected (Figure 6A).

**Figure 6:**
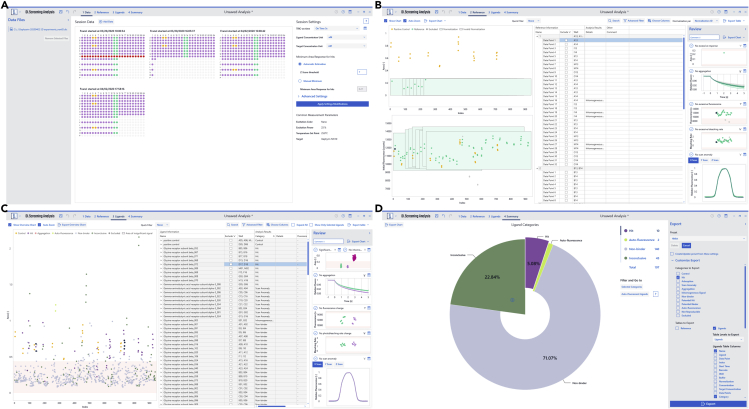
DI.ScreeningAnalysis single-dose screening (A) In the “data” section of the program, datasets can be selected and statistical parameters for hit identification (Z-score value) may be customized. (B) In the “reference” section, reference measurements may be manually inspected. (C) the “ligands” section allows to inspect each measured datapoint and manually exclude datapoints. (D) In the “summary” section, overviews are automatically generated and analyzed datasets may be exported.
36. On DI.ScreeningAnalysis’ data page, depending on the statistical variation of the references, the standard hit threshold of the 3-fold global standard deviation of references can be modified. In this assay we selected a z-score (i.e., multiplication factor) of 7 (Figure 6A, Problem 5).
37. We recommend a manual inspection of all references in an assay to identify reference samples that have an aggregation bias that was not noticed by the automatic algorithms of the software (Figure 6B). This manual inspection is easily done on the reference page in DI.ScreeningAnalysis. Such reference samples can be manually excluded.
38. The automatically sorted ligands can be manually investigated on the ligands page in DI.ScreeningAnalysis. Sorting of ligands into different categories (such as hit, non-binder or aggregation) by the software algorithms can be manually overwritten by the user if necessary (Figure 6C).
39. The batch data export on the summary page in DI.ScreeningAnalysis can now be used to export a list of ligands in user-selected categories for further investigation e.g., in a dose-response experiment (Figure 6D).

### Data analysis (dose responses)

**Timing: 30 min**

**Figure 7:**
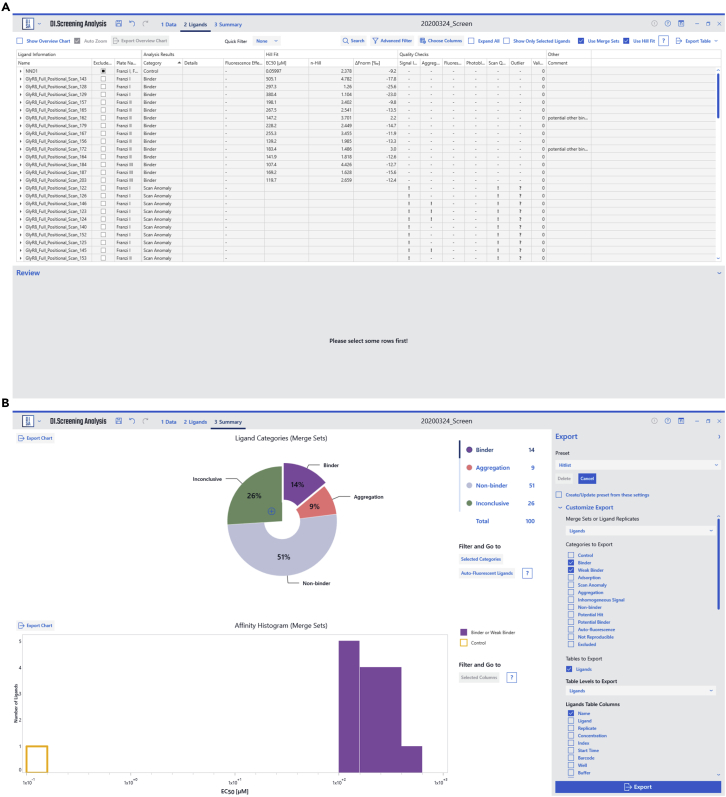
DI.ScreeningAnalysis dose-response (A) In the “ligands” section, the “merge sets” feature can be used to merge replicate datasets for analysis. Furthermore, the “use hill-fit” feature should be applied accordingly to the experimental setup. Additionally, individual datapoints may be manually inspected. (B) In the “summary” section, overviews are automatically generated, and analyzed datasets may be exported.
42. Depending on the assay conditions, select or deselect “use Hill fit” (Figure 7A).

***Note:*** The Hill fit is only recommended for competition assays or assays with known cooperativity. For direct binding of one ligand to one target molecule, the function is deselected and a law of mass action-based K_D_ fitting algorithm is commonly used.

40. In Di.Control, a dilution series ID is assigned to each well of a measured dataset. That way wells are grouped as one single dilution series for further analysis.
41. On the ligands page in DI.ScreeningAnalysis, select the “use merge sets” function that merges replicates of dilution series of the same ligand, depending on the ligand name. Dilution series are then shown as merged data sets that are approximated by a global fit to all datapoints (Figure 7A).

43. We recommend manual inspection of all ligands for smaller datasets to review software decisions, such as aggregation or ligand-induced quenching (Figure 7A).
44. Use the batch export on the summary page of DI.ScreeningAnalysis to export vector graphics of dose-response plots and raw-data values and fit parameters in a tabular format for further use (Figure 7B).

## Expected outcomes

### Single-dose screening

**Figure 8:**
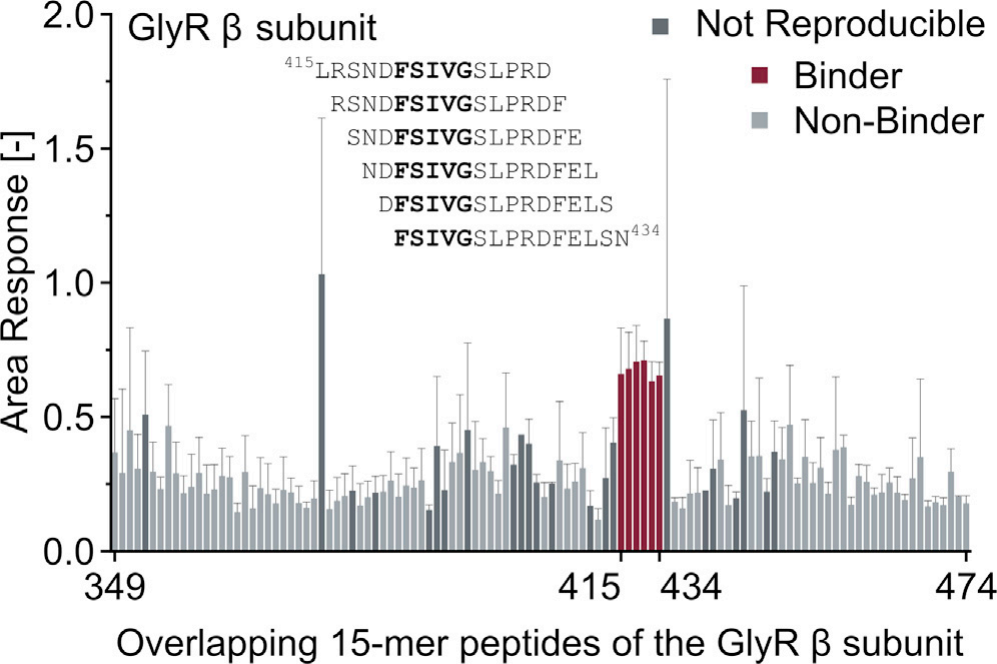
Expected results of a single-dose screening Peptide sequences of an overlapping peptide library. Identified binders are indicated; the core binding motif is highlighted in bold. Graph reprinted with permission from (Schulte et al., 2020). Averages of n = 1–6 measurements are shown with corresponding STDEV.

Single-dose screenings of overlapping peptide libraries identify binding hotspots in intrinsically disordered regions of proteins of interest (Figure 8). The quasi label-free displacement assay recapitulated the structurally resolved binding region of the glycine receptor β subunit binding to gephyrin E-domain (Maric et al., 2014). Binders, non-binders and inconclusive hits were automatically identified by the DI.ScreeningAnalysis software.

### Dose response affinity determination

**Figure 9:**
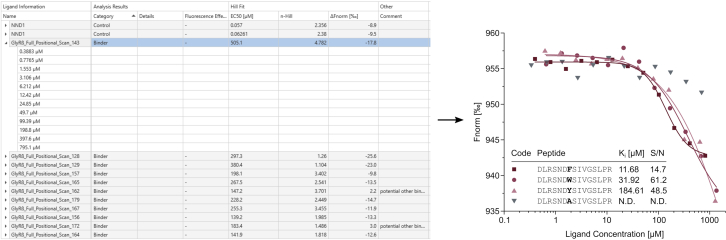
Expected results of a full-positional scan F_norm_ values are plotted against the respective concentration to determine the affinity of each peptide from a full positional scanning library. Graph reprinted with permission from Schulte et al. (2020).

Dose response assays allow determination of affinities and thereby elucidate binding requirements in single amino acid resolution (Figure 9). We conducted 12-point dose responses of a full-positional scanning library, corresponding to the core binding motif identified in the single-dose screening.

## Quantification and statistical analysis

In DI.ScreeningAnalysis, ligands are automatically assigned a category, depending on the data obtained for a particular ligand. The two main categories are “hits” and “non-binders” that are assigned based on the response amplitude measured. However, data obtained by Dianthus instruments contain additional information about a ligand, such as whether a ligand exhibits autofluorescence or induces target aggregation. Here, the ligand categories in single-dose screening and affinity screening are listed (Table 3).

**Table 3:** Ligand categories in single-dose screenings and dose-response TRIC affinity measurements

Single-dose screening		
Main category	Sub-category	Determining factor
Hit		None of the categories below apply and a significant TRIC signal response is observed
Non-binder		None of the categories below apply and no significant TRIC signal response is observed
Aggregation		The TRIC traces obtained for this ligand show an inhomogeneous pattern that is caused by aggregated or insoluble material (AIM) in the sample
Auto-fluorescence		The steady-state fluorescence for this target-ligand sample significantly exceeds the mean of the reference sample, indicating ligand autofluorescence or binding. User intervention and an additional control is required to discern binding from artifact
Inconclusive	Scan anomaly	The well scans show an aberrant shape that is caused by AIM accumulating at the well bottom
	Potential hit	Either the steady-state fluorescence for this target-ligand sample is significantly lower than the mean of the reference sample, or ligand-induced, time-dependent photobleaching is observed in the TRIC trace. These observations typically indicate spectral properties of the ligand e.g., causing an inner filter effect or a binding event that strongly alters the spectral properties of the fluorophore. User intervention and an additional control is required to discern binding from artifact
	Not reproducible	Replicates of the tested ligand do not yield the same category. For example, one replicate shows aggregation, while the other replicate does not
	Inhomogeneous signal	The measured TRIC responses for the replicates of a ligand strongly deviate, caused by liquid handling errors or a sample inhomogeneity, e.g., AIM
Affinity screening		
Main category	Sub-category	Determining factor
Binder	binder	None of the categories below apply and a significant binding amplitude is observed (difference between bound- and unbound state. Different F_norm_ thresholds for different selected on-times determine significance
	Weak binder	None of the categories below apply and a significant TRIC signal response is observed. The response amplitude is determined by the fit and the saturation level is determined from raw data and fit amplitude. Binders with a saturation of less than 50%
Non-binder		None of the categories below apply and no significant binding amplitude is observed
Aggregation		A significant number of wells within one merge set shows TRIC traces with an inhomogeneous pattern that is caused by AIM in the sample
Autofluorescence		The ligand shows a significant (more than +20% of the average) and ligand-dependent increase in initial fluorescence over a dilution series
Inconclusive	Scan anomaly	A significant number of wells within one merge set shows well scans with an aberrant shape caused by AIM accumulating at the well bottom
Inconclusive	Potential hit	The ligand shows a significant (more than −20% of the average) and ligand-dependent decrease in initial fluorescence over a dilution series or a ligand-dependent and significant change (20% increase or decrease from the average) in the obtained photobleaching rate.

## Limitations

The here presented approach for high-throughput peptide synthesis using chemically modified cellulose as a solid support requires the use of an acid-labile moiety for peptide cleavage. To this end, a Rink-amide group that yields peptides with an amide moiety at their C-terminus is routinely employed. 4-(4-hydroxymethyl-3-methoxyphenoxy)butyric acid (HMPB)-based strategies allow to obtain peptides with a terminal carboxyl group after acidic cleavage (Wang and Distefano, 2014), however, to the best of our knowledge, their application has not been exemplified in μSPOT format. Furthermore, the here presented peptide synthesis allows for the incorporation of unnatural aa derivates, yet, the maximum number of such building blocks in one synthesis is capped by the capacity of the rack for 10 mL tubes (48 slots).

Sequence-dependent fluorescence effects of peptides, such as autofluorescence or fluorescence quenching prevent their affinity determination via TRIC. However, such events are automatically identified by the DI.ScreeningAnalysis software and affected ligands are subsequently excluded from analysis.

## Troubleshooting

### Problem 1

Peptides exhibit aggregation during synthesis, resulting in a low peptide yield (step 3)

### Potential solution

Reducing the degree of Fmoc-β-Ala-OH functionalization by employing a functionalization mix with equal amounts of Fmoc-β-Ala-OH and Boc-Gly-OH can prevent aggregation during synthesis.

### Problem 2

Functionalized cellulose disks are removed from the 384-format tray by the needle of the MultiPep robot (step 34).

### Potential solution

Adjust the z-value of the robot needle under “tray” settings to avoid removal of the disks

### Problem 3

No pellet formation is observed after cleavage of the peptides form the solid support and precipitation of the peptide from the cleavage solution (steps 13 and 18).

### Potential solution

Perform longer centrifugation or an individual treatment of non-precipitated peptides is needed either by adding a drop of water or n-heptane, dependent on the hydrophilic or hydrophobic nature of peptide sequence.

### Problem 4

A large amount of precipitate is observed when centrifuging the 384-well Dianthus plate right before the measurement (step 31).

### Potential solution

If the protein of interest forms precipitates, the experimental conditions, such as the protein labeling strategy or the buffer composition, need to be adjusted.

### Problem 5

An unexpectedly high number of peptide ligands are identified as binders in a single dose screening (step 36)

### Potential solution

Adjust the Z-score threshold, by which the peptide ligands are classified as binders or enter a manual area minimum under “minimum area response for hits” in the DI.SA software.

## Resource availability

### Lead contact

Further information and requests for resources and reagents should be directed to and will be fulfilled by the lead contact, Hans Michael Maric (Hans.Maric@uni-wuerzburg.de).

### Materials availability

This study did not generate any new reagents.

### Data and code availability

Original data for single-dose and dose response assays in the paper (Figure 6, Figure 7, Figure 8, Figure 9) is available in (Schulte et al., 2020). There are restrictions to the availability of Software: The DI.ScreeningAnalysis and DI.Control Software can be licensed from Nanotemper Technologies GmbH. The MultiPep 2 – Celluspots Software can be licensed from CEM GmbH.
